# Use Patterns of Smartphone Apps and Wearable Devices Supporting Physical Activity and Exercise: Large-Scale Cross-Sectional Survey

**DOI:** 10.2196/49148

**Published:** 2023-11-22

**Authors:** Takeyuki Oba, Keisuke Takano, Kentaro Katahira, Kenta Kimura

**Affiliations:** 1 Human Informatics and Interaction Research Institute, National Institute of Advanced Industrial Science and Technology, Ibaraki, Japan

**Keywords:** mobile health, smartphone app, physical activity, wearable devices, telemedicine, wearable, wearables, mHealth, app, apps, use, usage, survey, cross sectional, cross-sectional, technology use, exercise

## Abstract

**Background:**

Physical inactivity is a global health issue, and mobile health (mHealth) apps are expected to play an important role in promoting physical activity. Empirical studies have demonstrated the efficacy and efficiency of app-based interventions, and an increasing number of apps with more functions and richer content have been released. Regardless of the success of mHealth apps, there are important evidence gaps in the literature; that is, it is largely unknown who uses what app functions and which functions are associated with physical activity.

**Objective:**

This study aims to investigate the use patterns of apps and wearables supporting physical activity and exercise in a Japanese-speaking community sample.

**Methods:**

We recruited 20,573 web-based panelists who completed questionnaires concerning demographics, regular physical activity levels, and use of apps and wearables supporting physical activity. Participants who indicated that they were using a physical activity app or wearable were presented with a list of app functions (eg, sensor information, goal setting, journaling, and reward), among which they selected any functions they used.

**Results:**

Approximately one-quarter (n=4465) of the sample was identified as app users and showed similar demographic characteristics to samples documented in the literature; that is, compared with app nonusers, app users were younger (odds ratio [OR] 0.57, 95% CI 0.50-0.65), were more likely to be men (OR 0.83, 95% CI 0.77-0.90), had higher BMI scores (OR 1.02, 95% CI 1.01-1.03), had higher levels of education (university or above; OR 1.528, 95% CI 1.19-1.99), were more likely to have a child (OR 1.16, 95% CI 1.05-1.28) and job (OR 1.28, 95% CI 1.17-1.40), and had a higher household income (OR 1.40, 95% CI 1.21-1.62). Our results revealed unique associations between demographic variables and specific app functions. For example, sensor information, journaling, and GPS were more frequently used by men than women (ORs <0.84). Another important finding is that people used a median of 2 (IQR 1-4) different functions within an app, and the most common pattern was to use sensor information (ie, self-monitoring) and one other function such as goal setting or reminders.

**Conclusions:**

Regardless of the current trend in app development toward multifunctionality, our findings highlight the importance of app simplicity. A set of two functions (more precisely, self-monitoring and one other function) might be the minimum that can be accepted by most users. In addition, the identified individual differences will help developers and stakeholders pave the way for the personalization of app functions.

## Introduction

### Background

Physical inactivity is an unresolved issue in modern society, despite its known risks to physical and mental health [1,2]. Mobile technology, including smartphone apps and wearable devices, is expected to become a game changer and has substantially impacted health care practices for both providers and recipients. Several behavior change techniques (BCTs) [3] are provided by apps in the form of self-help training, which is augmented by online (ambulatory) assessments of physiological status via wearable devices and smartwatches. Evidence supports the positive effects of mobile health (mHealth) interventions on physical activity (PA) [4-12], and a recent meta-analysis [13] suggested that activity trackers have a moderate-sized effect in improving PA, equating to an increase of 1800 steps per day and a reduction of 1 kg in body weight. Trials included in these (umbrella) meta-analyses implemented interventions not limited to self-monitoring (or just wearing an activity tracker); instead, activity trackers, combined with smartphone or web-based applications, offer more interactive BCT components, such as goals and planning, rewards and threats, social support, and gamification [9].

Regardless of the success of mHealth tools and interventions, there are important evidence gaps in the literature; that is, it remains largely unknown who uses what app functions and which functions are associated with PA [14]. In recent years, a large number of health care apps have appeared on the market, many of which have complex multifunctionality to cover different needs and provide person-centered care [15]. Indeed, the amount of content and number of functions are found to be predictive of the overall quality of PA apps [16], as well as the efficacy of an intervention [17]; however, users tend to consider an app valuable when it is simple and intuitive to use [18]. An analysis of commercial health care apps [18] identified 12 representative features and characteristics (ie, export data, gamification, general education, plans or orders, reminders, community forums, social media, address symptoms, tailored education, tracking, cost, and usability), among which the export of data, usability, and cost were associated with users’ positive ratings. A qualitative study on users’ perceptions of apps [19] found that people typically like the tracking feature (eg, monitoring step counts) in their apps because this type of self-monitoring increases their awareness, and feedback on the tracked data helps them observe their progress. A cross-sectional survey of Chinese app users [20] identified typical health app users to be women and in a higher self-rated social class; the most prevalent types of apps were those that provide health information, track vital signs (eg, steps or heart rate), and provide health and medical reminders. A similar analysis was conducted in Saudi Arabia [21], showing that daily step counting and ovulation tracking (among women) were the most prevalent functions. Analyses of representative samples of Dutch [22] and US populations [23] suggested that mHealth app users are generally younger and more educated, and have higher levels of eHealth literacy skills than nonusers, although the profile of app users varies largely across different types of apps (eg, for fitness, nutrition, sleep, and mindfulness).

### Objectives

In short, previous studies have suggested that gender, age, and education level are robust predictors of mHealth app use, and tracking (eg, step counts) appears to be the most prevalent function. However, heterogeneity persists due to the types of apps and demographic profiles of app users. A systematic investigation is warranted to clarify the associations between user profiles and the use patterns of individual app functions that help increase PA. Thus, this study aims to determine the prevalence of commercial PA apps in a community sample. We were specifically interested in sociodemographic differences between app users and app nonusers, which app functions would be used most frequently, which app functions would be associated with increased levels of PA, and which user profiles (eg, gender and age) would be predictive of the use of the app functions associated with increased levels of PA.

## Methods

### Participants and Procedure

We analyzed the data of 20,573 Japanese-speaking web-based panelists (n=10,701 women; mean age 52.7, SD 17.8 years) sampled from a survey company’s database in which >1.3 million inhabitants were registered as potential participants. Participants were recruited with appropriate weights that reflected the demographic composition (eg, place of residence) across the country. We did not use any inclusion criteria, except for age (≥18 years). The sample size was determined arbitrarily, and the overarching project was published elsewhere [24]. In short, this project consisted of a series of web-based surveys administered on three different occasions, each targeting different aspects of PA behavior and health. The first wave of the survey covered regular PA levels as well as motivational and environmental factors that potentially influence PA behavior, such as decisional balance, self-efficacy, and social support. The second wave, which is reported in this paper, specifically focuses on mHealth technology use (ie, apps and wearable devices). The third wave involved past and current physical and mental disorders (not reported). The second and third wave of surveys were administered in the same week (early 2023), approximately 2 months after the first wave of surveys.

### Ethical Considerations

Participants provided informed consent in the first wave. At the end of each survey, they were compensated with a voucher for web-based shopping (value: approximately US $0.31). The study protocol was approved by the Ethics Committee of the National Institute of Advanced Industrial Science and Technology (approval ID 2022-1279). We adhered to the STROBE (Strengthening the Reporting of Observational Studies in Epidemiology) Statement [25] when conducting and reporting this study.

### Measures

#### Stage of Change Questionnaire

PA readiness was assessed using the Japanese version [26,27] of the stage of change (SoC) questionnaire [28,29]. Each participant was classified into one of five stages (ie, precontemplation, contemplation, preparation, action, and maintenance) according to their responses to the following items: “I currently do not exercise and do not intend to start exercising in the future” (precontemplation); “I currently do not exercise but I am thinking about starting to exercise in the next six months” (contemplation); “I currently exercise some, but not regularly” (preparation); “I currently exercise regularly, but have only begun doing so within the last six months” (action); and “I currently exercise regularly and have done so for longer than six months” (maintenance). Regular exercise was operationalized as exercising twice or more per week for 20 minutes or longer, which was explicitly stated to the participants.

#### International PA Questionnaire–Short Form

Average weekly PA levels were assessed using the International Physical Activity Questionnaire–Short Form (IPAQ-SF) [30,31]. The IPAQ-SF includes the following three PA dimensions: (1) walking, (2) moderate-intensity activity, and (3) vigorous-intensity activity (sedentary time was not used for our analyses). Participants reported the number of days and duration spent on each dimension of PA over an average week. The reported weekly minutes of PA were transformed into metabolic equivalent tasks (METs per hour), which allowed us to explore how many participants adhered to the Japanese public health guidelines for PA: 23 METs per hour per week for adults [32]. We also coded the IPAQ-SF responses in a categoric manner: inactive, minimally active (≥3 days of ≥20 minutes of vigorous activity or equivalent), and health-enhancing physical activity (HEPA) active (≥3 days of vigorous activity achieving 25 METs per hour per week or equivalent) [33].

#### Use of Apps and Wearables

Participants were first asked to indicate whether they used any apps to support PA or exercise (in the first-wave survey). Those who responded affirmatively were invited to the second-wave survey, where they provided the following information (see Multimedia Appendix 1 for details): (1) the names of apps in use, (2) what sensors or wearable devices were connected to the apps (if any), (3) how long they had been using the app that they were using most frequently (less than a week to more than a year), (4) how frequently they were using the app (less than once per month to multiple times per day), (5) sources of information about the app (eg, preinstalled on a smartphone or learned from a family, friend, or health specialist), and (6) which functions of the app they were using. For item 6, a list of 41 app functions was presented to each participant (eg, sensor information, goal setting, and journaling), and participants selected any applicable function. This list was generated by the first author based on published studies in the literature [15,18]. The list was reviewed by four researchers using different PA apps and devices.

### Statistical Analyses

First, we tested the prevalence of apps and individual functions and explored the demographic differences between app users and app nonusers (eg, gender, age, education, income, and levels of PA and SoC). Second, we visualized the use patterns of the app functions in the form of a network. We were specifically interested in app functions that were often used together, and these co-occurrences were represented by edges in the network. The backbone algorithm was used to select meaningful edges in the network. In this algorithm, each edge weight (ie, the co-occurrence frequency) is normalized by the strength of the connected nodes (ie, the sum of the edge weights of each node), which is then statistically tested (α=.05) under the assumption that the normalized edge weights are uniformly distributed [34]. Third, multinomial logistic regression analysis was conducted to examine the associations between self-reported PA levels and app functions. A series of logistic regression analyses were conducted to explore the demographic variables that were predictive of the use of each function. All analyses were performed using R (version 4.2.2; R Foundation for Statistical Computing) with the *backbone* package [35], which provided a *disparity* function for network edge selection.

## Results

### Prevalence of PA Apps

Among 20,573 individuals, 5030 (24.4%) reported using one or more PA apps. These app users were invited to participate in the second-wave survey, in which 4465 participants completed questionnaires concerning PA app use. The most frequently used apps were the iOS health app (n=1239, 27.8%), Google Fit (n=910, 20.4%), dHealthcare (n=891, 20%), and Trima (n=1026, 23%). Most app users (n=3140, 70.3%) had been using a PA app longer than 6 months (n=2583, 57.8%), and most used the app once or more per day (n=3218, 72.1%). App users reported that they started to use the app because it was installed when they purchased their smartphones or tablets (n=1589, 35.6%) or they learned about the app on the internet or social media (n=1387, 31.1%), or from someone close to them, such as family members, friends, acquaintances, or colleagues (n=1208, 27.1%).

### Demographics of App Users Versus App Nonusers

Table 1 shows the demographic characteristics of the app users and app nonusers. To explore the demographic differences between app users and app nonusers, a logistic regression analysis was performed with app users (vs app nonusers) as the outcome (see also Table S1 in Multimedia Appendix 2). The results showed that, compared to app nonusers, app users were younger, were more likely to be men, had larger BMI scores, had higher levels of education (university or above), were more likely to have a child and job, and had a higher household income. App users were more active with a median PA level of 30.8 METs per hour per week (vs 13.6 METs/hour/week among app nonusers), indicating that most of them adhered to the national health recommendation (23 METs/hour/week). This tendency is also endorsed by the SoC distribution: almost half of app users were in the maintenance stage (2065/4465, 46.2%; ie, having exercised regularly for more than 6 months), which is more prevalent than with app nonusers (4327/15,543, 27.8%).

**Table 1.** Demographic statistics of app users and app nonusers.

Variable		App users *(*n=4465)	App nonusers (n=15,543)	Odds ratio^a^ (95% CI)		*P* value
Age (years), mean (SD)		50.70 (17.36)	53.48 (17.67)	N/A^b^		N/A
**Age group (years)^c^, n (%)**						
	<30	655 (14.7)	1953 (12.6)	N/A		N/A
	30-44	1073 (24.0)	3139 (20.2)	0.870 (0.768-0.987)		.03
	45-59	1135 (25.4)	3856 (24.8)	0.736 (0.648-0.835)		<.001
	≥60	1602 (35.9)	6595 (42.4)	0.570 (0.499-0.651)		<.001
Women, n (%)		1932 (43.3)	8461 (54.4)	0.832 (0.769-0.900)		<.001
BMI, mean (SD)		22.34 (3.69)	22.08 (3.71)	1.018 (1.008-1.028)		<.001
Married, n (%)		2899 (64.9)	9719 (62.5)	1.049 (0.952-1.157)		.34
One or more children, n (%)		2772 (62.1)	9585 (61.7)	1.156 (1.047-1.277)		.004
**Education level, n (%)**						
	Middle school	78 (1.7)	426 (2.7)	N/A		N/A
	High school	1135 (25.4)	5081 (32.7)	1.159 (0.898-1.513)		.27
	College or vocational school	892 (20.0)	3689 (23.7)	1.262 (0.974-1.654)		.08
	University or above	2314 (51.8)	6230 (40.1)	1.528 (1.186-1.991)		.001
	Others	46 (1.0)	117 (0.8)	2.265 (1.453-3.509)		<.001
Job, n (%)		3058 (68.5)	8964 (57.7)	1.277 (1.170-1.395)		<.001
**Household income (¥ ^d^), n (%)**						
	<3 million	827 (18.5)	3467 (22.3)	N/A		N/A
	3-5 million	1012 (22.7)	3869 (24.9)	0.919 (0.824-1.026)		.13
	5-7 million	753 (16.9)	2310 (14.9)	1.030 (0.910-1.166)		.64
	7-10 million	700 (15.7)	1804 (11.6)	1.122 (0.984-1.279)		.09
	≥10 million	559 (12.5)	1076 (6.9)	1.401 (1.213-1.618)		<.001
	No answer	614 (13.8)	3017 (19.4)	0.793 (0.702-0.896)		<.001
Physical activity (METs^e^/h/wk), median (IQR)		30.8 (12.0-62.2)	13.6 (1.7-34.8)	N/A		N/A
**Physical activity, n (%)**						
	Inactive	1257 (28.2)	7664 (49.3)	N/A		N/A
	Minimally active	1762 (39.5)	5362 (34.5)	1.579 (1.448-1.723)		<.001
	HEPA^f^ active	1446 (32.4)	2517 (16.2)	2.132 (1.925-2.362)		<.001
**Stage of change, n (%)**						
	Precontemplation	390 (8.7)	3943 (25.4)	N/A		N/A
	Contemplation	728 (16.3)	3768 (24.2)	1.875 (1.642-2.143)		<.001
	Preparation	982 (22.0)	2915 (18.8)	2.897 (2.547-3.302)		<.001
	Action	300 (6.7)	590 (3.8)	3.676 (3.067-4.405)		<.001
	Maintenance	2065 (46.2)	4327 (27.8)	3.628 (3.199-4.122)		<.001
App functions in use, median (IQR)		2 (1-4)	N/A	N/A		N/A

a Odds ratios calculated in the logistic regression predicting app versus app nonusers.

b N/A: not applicable.

c Age was treated as a categorical predictor in the logistic regression with age <30 years as the reference.

d A currency exchange rate of ¥140=US $1 is applicable.

e MET: metabolic equivalent task.

f HEPA: health-enhancing physical activity.

We also explored the demographic characteristics per stage of change (Table 2). Similar to published studies in the literature (see Marshall and Biddle [36] for meta-analytic evidence), the most frequent stage was maintenance. Participants identified at the maintenance stage were typically older (aged ≥60 years), married, and highly educated, and had a child, job, and high income.

**Table 2.** Demographics per stage of change.

		Precontemplation(n=4391), n (%)	Contemplation(n=4641), n (%)	Preparation(n=4006), n (%)	Action(n=945), n (%)	Maintenance(n=6590), n (%)	Chi-square (*df*)	*P* value
**Gender**							238.6 (4)	<.001
	Men	2123 (48.3)	1789 (38.5)	2017 (50.3)	457 (48.4)	3486 (52.9)		
	Women	2268 (51.7)	2852 (61.5)	1989 (49.7)	488 (51.6)	3104 (47.1)		
**Age group (years)**							994.9 (12)	<.001
	<30	605 (13.8)	782 (16.8)	557 (13.9)	222 (23.5)	624 (9.5)		
	30-44	974 (22.2)	1218 (26.2)	861 (21.5)	249 (26.3)	1026 (15.6)		
	45-59	1230 (28.0)	1308 (28.2)	1018 (25.4)	198 (21.0)	1336 (20.3)		
	>60	1582 (36.0)	1333 (28.7)	1570 (39.2)	276 (29.2)	3604 (54.7)		
**Marital status**							71.3 (4)	<.001
	Yes	2581 (58.8)	2861 (61.6)	2574 (64.3)	574 (60.7)	4364 (66.2)		
	No	1810 (41.2)	1780 (38.4)	1432 (35.7)	371 (39.3)	2226 (33.8)		
**One or more children**							116.5 (4)	<.001
	Yes	2554 (58.2)	2732 (58.9)	2488 (62.1)	523 (55.3)	4375 (66.4)		
	No	1837 (41.8)	1909 (41.1)	1518 (37.9)	422 (44.7)	2215 (33.6)		
**Education level**							177.0 (16)	<.001
	Middle school	167 (3.8)	96 (2.1)	96 (2.4)	24 (2.5)	130 (2.0)		
	High school	1510 (33.4)	1407 (30.3)	1241 (31.0)	258 (27.3)	1973 (29.9)		
	Some college	1009 (23.0)	1231 (26.5)	883 (22.0)	218 (23.1)	1374 (20.8)		
	College and above	1662 (37.9)	1876 (40.4)	1739 (43.4)	436 (46.1)	3077 (46.7)		
	Others	43 (1.0)	31 (0.7)	47 (1.2)	9 (1.0)	36 (0.5)		
**Job**							207.2 (4)	<.001
	Yes	2666 (60.7)	3040 (65.5)	2558 (63.9)	609 (64.4)	3527 (53.5)		
	No	1725 (39.3)	1601 (34.5)	1448 (36.1)	336 (35.6)	3063 (46.5)		
**Household income (¥** ^**a**^**)**							190.9 (20)	<.001
	<3 million	1101 (25.1)	948 (20.4)	825 (20.6)	191 (20.2)	1369 (20.8)		
	3-5 million	1054 (24.0)	1111 (23.9)	942 (23.5)	228 (24.1)	1674 (25.4)		
	5-7 million	628 (14.3)	743 (16.0)	665 (16.6)	157 (16.6)	950 (14.4)		
	7-10 million	439 (10.0)	609 (13.1)	527 (13.2)	131 (13.9)	877 (13.3)		
	≥10 million	291 (6.6)	318 (6.9)	315 (7.9)	69 (7.3)	699 (10.6)		
	No answer	878 (20.0)	912 (19.7)	732 (18.3)	169 (17.9)	1021 (15.5)		

a A currency exchange rate of ¥140=US $1 is applicable.

### App Functions: Prevalence and Associations Between Functions

**Figure 1.**
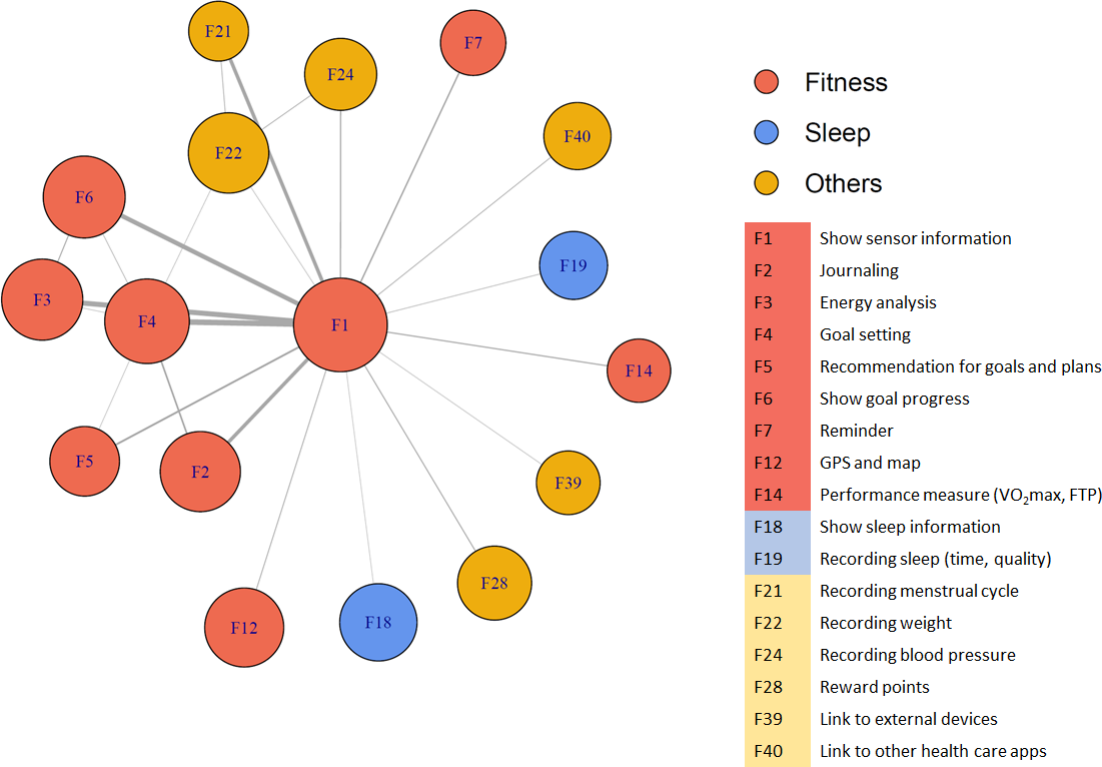
Use patterns of app functions. FTP: functional threshold power; VO_2_max: maximum oxygen consumption.

The 4465 total app users reported that apps were typically synchronized to a step counter or pedometer (n=3167, 70.9%), GPS and map functions (n=1527, 34.2%), body scale (n=882, 19.8%), and heart rate monitor (n=619, 13.9%). These sensors are also implemented in smartwatches, which were used by 20.8% (n=928) of the app users. Table 3 shows the 10 most frequently used apps or sensor functions. More than half of the app users monitored sensor information (eg, step counts, heart rates, and skin temperature); some also used functions to support goal setting and visualize goal progress. Figure 1 shows how each function is used together with other functions. Sensor information is typically used with one other function (eg, goal setting, goal progress, and recording the menstrual cycle), and participants used a median of 2 (IQR 1-4) different functions within an app (Table 1). It is uncommon for participants to use 5 or more functions regardless of the multifunctionality of apps on the market.

**Table 3.** The 10 most frequently used app functions.

App function	Participants, n (%)
Show sensor info	2580 (57.78)
Goal setting	1203 (26.94)
Show goal progress	965 (21.61)
Energy analysis	903 (20.22)
Weight recording	845 (18.92)
Journaling	831 (18.61)
GPS/map	756 (16.93)
Show sleep info	655 (14.67)
Reward points	496 (11.11)
Blood pressure recording	419 (9.38)

### App Functions and PA

Table 4 shows the results of the multinomial logistic regression analysis where PA (contrasts: inactive vs minimally active; inactive vs HEPA active) was predicted by the 10 most frequently used app functions. Only 2 app functions (ie, sensor information and goal setting) were identified as significant predictors to distinguish between inactive and minimally active individuals. On the other hand, 6 app functions (ie, sensor information, goal setting, goal progress, journaling, GPS/map, and energy analysis) were found informative in predicting HEPA-active individuals. These functions are typically designed and implemented to target PA. Functions that are not significantly related to PA have other primary health targets, such as sleep and nutrition.

**Table 4.** Multinomial logistic regression predicting physical activity level as a categorical variable for all app users (n=4465).

Variables^a^		Estimate (SE)		*Z*	*P* value	Odds ratio (95% CI)	
**Dependent variable contrast: inactive vs minimally active**							
	Show sensor info	0.331 (0.077)		4.308	<.001	1.393 (1.198-1.620)	
	Goal setting	0.527 (0.095)		5.525	<.001	1.693 (1.405-2.041)	
	Show goal progress	0.154 (0.102)		1.508	.13	1.166 (0.955-1.425)	
	Energy analysis	0.192 (0.103)		1.861	.06	1.212 (0.990-1.485)	
	Weight recording	0.139 (0.108)		1.284	.20	1.149 (0.930-1.419)	
	Journaling	0.114 (0.104)		1.096	.27	1.120 (0.914-1.373)	
	GPS/map	0.018 (0.107)		0.166	.87	1.018 (0.826-1.255)	
	Show sleep information	0.072 (0.116)		0.621	.54	0.930 (0.741-1.168)	
	Reward points	0.215 (0.124)		1.738	.08	1.240 (0.973-1.579)	
	Blood pressure recording	0.246 (0.147)		1.671	.10	1.279 (0.958-1.707)	
**Dependent variable contrast: inactive vs HEPA**^**b**^ **active**							
	Show sensor info	0.278 (0.081)		3.435	.001	1.320 (1.127-1.546)	
	Goal setting	0.480 (0.099)		4.830	<.001	1.617 (1.330-1.964)	
	Show goal progress	0.226 (0.106)		2.144	.03	1.254 (1.020-1.542)	
	Energy analysis	0.265 (0.107)		2.491	.01	1.304 (1.058-1.607)	
	Weight recording	0.104 (0.113)		0.918	.36	1.109 (0.889-1.384)	
	Journaling	0.470 (0.103)		4.540	<.001	1.600 (1.306-1.960)	
	GPS/map	0.306 (0.107)		2.847	.004	1.357 (1.100-1.675)	
	Show sleep information	0.068 (0.119)		0.573	.57	1.070 (0.848-1.350)	
	Reward points	0.106 (0.131)		0.807	.42	1.111 (0.860-1.437)	
	Blood pressure recording	0.067 (0.156)		0.430	.67	1.069 (0.788-1.452)	

a The independent variable was app function

b HEPA: health-enhancing physical activity.

For the 6 PA-related functions (Table 4), we further examined their associations with demographic variables and explored the characteristics of users of each function. The results of the logistic regression analyses (see Table S2 in Multimedia Appendix 2) suggested that older people (aged ≥60 years) were more likely to use goal management functions (goal setting: odds ratio [OR] 1.33, 95% CI 1.04-1.70; *P*=.02; goal progress: OR 2.23, 95% CI 1.70-2.96; *P*<.001), and middle-aged people preferred the GPS and map functions (OR 1.40, 95% CI 1.05-1.87; *P*=.02). Sensor information (OR 0.84, 95% CI 0.73-0.96; *P*=.01), journaling (OR 0.80, 95% CI 0.67-0.96; *P*=.02), and GPS (OR 0.59, 95% CI 0.49-0.71; *P*<.001) were used more frequently by men than by women. Education level (university or above) predicted the use of sensor information (OR 1.17, 95% CI 1.01-1.36; *P*=.04), journaling (OR 1.38, 95% CI 1.13-1.68; *P*=.002), and energy analysis (OR 1.29, 95% CI 1.07-1.56; *P*=.009). Household income was a significant predictor of most PA-related functions; typically, people with the highest income (≥¥10 million/year, US $71,400/year) used PA-related functions.

## Discussion

### Principal Findings

We investigated the use patterns of apps and wearables that support PA and exercise among Japanese-speaking adults. Our results replicated the characteristics of app users found in other countries; that is, mHealth app users are generally younger and more educated, and have higher social and economic statuses than app nonusers [20,21,23]. These user characteristics may be generalizable to wearable activity trackers, as studies on the US populations suggest that age, gender, ethnicity, income, and health conditions are associated with device use [37-39]. A notable difference is that male users were more prevalent than female users in our data, contrasting the previous findings on mHealth apps in general. An investigation of the Dutch population suggested that this may be a unique pattern for fitness apps; that is, fitness apps are more frequently used by men, while apps concerning nutrition and self-care are more prevalent among women [22]. Indeed, this study targeted PA apps exclusively, which may explain the identified gender differences.

Another important finding is that people typically use no more than 4 functions within an app, and the most common pattern is to use sensor information (ie, self-monitoring in the BCT taxonomy) and one other function, such as goal setting and reminders. The amount of content and number of functions are suggested to be associated with users’ ratings [16]; in addition, effective interventions for weight loss implemented 3-6 times more BCTs than ineffective interventions [17]. These previous findings support the current trend of app development, that is, increasing multifunctionality and enriching the content of apps. However, another content analysis on apps and user ratings suggested that app users tend to identify apps as valuable when they are simple and intuitive to use [18]. Furthermore, a recent meta-analysis [15] failed to find an association between the number of implemented BCTs and the efficacy of mHealth interventions. Our findings highlight the importance of simplicity (rather than complexity and richness of app functions) because few of the implemented functions appear to reach individual users.

There is robust evidence that self-monitoring is useful for increasing PA and improving dietary behavior [40,41]. In addition, Michie and colleagues [42] found that interventions that combined self-monitoring with at least one other BCT were more effective than other interventions. Our findings are in line with this “self-monitoring plus one” principle, as sensor information was the most frequently used function, and this was often used together with one other function (participants typically used only two different functions within an app). Taken together, it may be possible that the minimum set of effective interventions would be to provide self-monitoring (displaying sensor information; eg, steps and heart rate) and one other BCT function (eg, goal setting or rewards; possibly personalized according to the preferences of users). Such a minimal approach may be appreciated for its simplicity and usability, leading to a better user experience and better health outcomes.

We also observed significant individual differences in the use of each app function. A series of logistic regressions identified that demographic variables (ie, age, gender, education level, and household income) are predictive of the use of app functions associated with regular PA levels (ie, sensor information, goal setting, goal progress, journaling, GPS and map, and energy analysis). It is not surprising that individuals with different backgrounds need different app functions. Indeed, previous findings have provided evidence for educational, age, and gender differences in the use of mHealth devices and apps [22,23,43]; however, McCully et al [44] reported no gender differences in the use of the internet for diet, weight, and PA. Carrol and colleagues [23] argued that educational attainment reflects skills and confidence with the use of devices and possibly social norms related to the perceived value (of staying healthy). The gender differences identified in this study are overall in line with other findings about the Dutch population; men appear to prefer the functions directly relevant to fitness and exercise (ie, sensor information, journaling, and GPS/map). However, when it comes to general health apps (for diet, nutrition, and self-care), the literature shows that women are more dominant users [22,43]. Another study suggested that women often report external goals of PA (eg, weight loss and toning), whereas men tend to engage in PA for enjoyment [45]. Such intrinsic motivation toward PA among men may facilitate the use of PA-related app functions.

Age is also an important predictor of app use—in general, older people do not use mHealth services [46]. Our results overall replicated this tendency, which may point to digital divides among an older population. However, we did not assess ownership of smart devices or eHealth literacy, which prevented us from exploring how prior knowledge and experiences with mHealth tools influenced actual app use. Interestingly, older app users were more likely to use goal management functions than younger users. These results might indicate that older people are more sensitive to their PA goals and progress, which are often linked to risks of chronic diseases. The Japanese Ministry of Health, Labour and Welfare [32] explicitly defines the step goals (eg, 8000 steps) to achieve each day in the context of lifestyle changes for preventing noncommunicable diseases. It is known that apps for self-care or measuring vitals are typically used by older adults [22], and older individuals may be more concerned with health/disease markers (eg, PA levels or blood pressure) to be monitored on mHealth apps.

### Limitations

Several important limitations may affect the interpretation of the results. The cross-sectional nature of this study limits our ability to infer causality. It is not yet clear whether the use of a particular app function increases PA levels or whether individuals who are already active prefer to use the app function. In addition, the generalizability of our findings needs to be tested, as we exclusively targeted Japanese-speaking adults, and its app markets differ from those in other countries. Some of the most prevalent apps (eg, dHealthcare and Trima) limit their services to users living in Japan, whereas apps that are common in the West (eg, MyFitnessPal) were less prevalent in our sample. Additionally, it may be an important direction to explore a wider range of cultural and sociodemographic differences [47], as this study exclusively focused on the population living in a high-income country with a monotonous cultural background. Another important limitation is that we did not consider factors that motivated users to continue using PA apps in our analyses. Research has shown that the retention rate of commercial health care apps is extremely low [48], and different users have different motivations to maintain the use of an app [49,50], such as adjusting default settings to one’s own needs and abilities, socializing, and competition. Additionally, it is important to consider users’ willingness to share their data from mHealth tools with providers as well as peers and family, which is key when implementing mHealth services as a meaningful intervention [51,52]. Future studies should explore the motivational aspects of app use to clarify why and how people use their PA apps.

### Conclusion

We provide empirical evidence on the use patterns of commercial apps and wearables as well as the individual functions implemented with the apps. Overall, our findings are in line with those of previous studies (eg, app users tend to be younger, have a higher income, and have higher education than app nonusers); however, our results showed unique associations between particular demographic variables and specific app functions (eg, sensor information, journaling, and GPS are more frequently used by men than women). These individual differences will help pave the way for the personalization of app functions, leading to the optimization and improved efficiency of mHealth interventions for promoting PA.

## Supplementary material

10.2196/49148 Multimedia Appendix 1 The questionnaire for uses of apps and wearables supporting physical activity.

10.2196/49148 Multimedia Appendix 2 Additional logistic regression analyses.

## Abbreviations

BCT: behavior change technique
HEPA: health-enhancing physical activity
IPAQ-SF: International Physical Activity Questionnaire–Short Form
MET: metabolic equivalent task
mHealth: mobile health
OR: odds ratio
PA: physical activity
SoC: stage of change
STROBE: Strengthening the Reporting of Observational Studies in Epidemiology
