# Supplementary material for: Use Patterns of Smartphone Apps and Wearable Devices Supporting Physical Activity and Exercise: Large-Scale Cross-Sectional Survey
Source: JMIR Mhealth Uhealth. 2023 Nov 22;11:e49148. doi: 10.2196/49148 (PMC10690103; doi:10.2196/49148)
Supplement: Multimedia Appendix 1 [file mhealth-v11-e49148-s001.docx]

# Appendix

The Questionnaire for Uses of Apps and Wearables Supporting Physical Activity

Instructions: Many apps and mobile devices have been developed to support daily exercise and physical activity. This survey asks you about your use of such apps and mobile devices.

1. Do you use smartphone apps (including tablets) to support your exercise? Any purpose of use is acceptable (e.g., diet, fitness, health management). However, apps that "only" record weight, menstrual cycle, or food management are not included.

2. If you selected "Yes," please answer the following questions. Which application are you currently using? Please select all that apply from the following items.

- Google Fit
- Healthcare (iPhone)
- d healthcare
- Trima
- MyFitnessPal
- Strava
- [Other commercial apps available in Japan follow]

3. For any of the apps selected in Item 2, we would like to know what kind of sensors the apps are synchronized/connected with. Please select all that apply from the list below. Only those apps that automatically collect and record information (e.g., your weight is uploaded and displayed on an app via network whenever you use your weighing scale), but not those that manually input information (e.g., you read the scale value yourself and manually input it into the app). Please answer this question not only for built-in sensors of a smartphone (e.g., step-counting function), but also for external devices/sensors that are synchronized to your smartphone (e.g., heart rate monitors, skin thermometers). If you use a smartwatch, please indicate the name and model, and select all applicable sensor functions in use (e.g., if you use Fitbit Sense 2, please select smartwatch, indicate Fitbit Sense 2, and select pedometer, GPS, acceleration, heart rate, etc.). You do not need to select any sensors/functions that you are not using.

- Smartwatch (e.g., Fitbit Sense, Garmin Fenix, Apple watch, G-shock Pro Trek, HUAWEI WATCH FIT, Polar, Suunto): Please indicate the product/model you are using (free description; if you choose a smartwatch, please indicate the model you are using) If you selected smartwatch, enter the required information.
- Pedometer/step counter
- GPS and/or location-data recording
- Accelerometer/actigraph
- Heart rate monitor
- Blood pressure monitor
- Blood oxygen level monitor
- Body weight meter
- Thermometer/skin thermometer
- Other sensors (speedometer, power meter, etc.; open ended question)

4. Among the apps you selected in Item 2, which app do you use most often? Please indicate the name of that app below.

5. Regarding the app that you use most frequently, how long have you been using this app? Please select the option that best describes your use of the app.

- < 1 week
- ≥ 1 week, < 1 month
- ≥ 1 month, < 3 months
- ≥ 3 months, < 6 months
- ≥ 6 months, < 1 year
- ≥ 1 year

6. Regarding the app that you use most frequently, how often do you use it? Please select the option that best describes your use of the app.

- Several times a day or more
- About once a day
- Once every 2 or 3 days
- About once a week
- About once a month
- Less often than that

7. We are interested in how you started using the app that you use most frequently. Please select all the options that apply to you.

- Installed when I purchased my smartphone/tablet
- Told by someone close to me, such as a family member, friend, acquaintance, or colleague.
- Told by an expert (doctor, medical professional, gym/sports trainer)
- Learned about it on TV
- Learned about it on the Internet or social media (Facebook, Twitter, Instagram).
- Others (Free answer).

8. Which of the following functions do you use? Please select only those functions that you use, and do not select functions that you do not use but are provided by the apps. If you use external devices or sensors such as smartwatches, please include the functions provided by those devices. If you use multiple functions, please select all applicable ones.

--- Fitness-related

1. Displaying and graphing statistics from sensors (e.g., step count, distance traveled, heart rate) [# Showing sensor information]
2. Journal (e.g., notes on steps and other fitness/training-related items to be recorded or entered manually) [# Journal]
3. Estimation and analysis of energy consumption [# Energy analysis]
4. Setting personal goals (e.g., target number of steps per day, target energy expenditure) [# Goal setting]
5. Recommendations for personal goals and training plans (e.g., displaying exercise recommendations)
6. Displaying goal progress and status [# Showing goal progress]
7. Reminders
8. Training and workout planning
9. Route/course creation and route-search functions (e.g., for running and cycling)
10. Leaderboard (compare/compete with other users)
11. Create and participate in races, events, and competitions (including assistance in registering for a public marathon)
12. GPS and map [# GPS/map]
13. Recording water intake/supplementation
14. Performance and cardiopulmonary measurement (VO2max, FTP, etc.)

--- Diet-related

1. Daily meal record
2. Barcode scanner (meal record)
3. Analysis of calorie intake

--- Sleep-related

1. Displaying sleep duration and quality (graph or score automatically calculated by the app) [# Showing sleep information]
2. Sleep time and sleep quality record (sleep time, wake-up time, etc., to be entered by the user)

--- Others

1. Relaxation, mindfulness
2. Recording menstrual cycle
3. Weight recording (manual input, not automatically synchronized to the application) [# Weight recording]
4. Recording of blood glucose level (manual input)
5. Recording blood pressure (manual input) [# Blood-pressure recording]
6. Medication management and recording
7. Safety management functions (e.g., traffic accident notification)
8. Game elements (e.g., catching and defeating monsters, acquiring in-game items)
9. Reward points/shopping coupons (which can be used online) [# Reward points]
10. Virtual currency (e.g., Bitcoin)
11. Badge and trophy functions
12. Provision of health-related information (e.g., newsletters)
13. Prediction of future health and body shape
14. Privacy settings (e.g., restrictions on public scope and access restrictions)
15. Profile settings and modifications
16. Push notifications (e.g., exercise reminders, notifications when goals are met)
17. Scheduling functionality
18. Help function
19. Networking with friends and other users (e.g., messages, chats, groups)
20. Linkage and synchronization with devices and sensors outside the smartphone
21. Linkage and synchronization with other healthcare apps
22. Linkage with other apps (e.g., SNS or chat)
23. Other (open-ended)

Note. [# items] are the functions most frequently used in the current sample.
