# Supplementary material for: Use Patterns of Smartphone Apps and Wearable Devices Supporting Physical Activity and Exercise: Large-Scale Cross-Sectional Survey
Source: JMIR Mhealth Uhealth. 2023 Nov 22;11:e49148. doi: 10.2196/49148 (PMC10690103; doi:10.2196/49148)
Supplement: Multimedia Appendix 2 [file mhealth-v11-e49148-s002.docx]

**Table S1**

Logistic Regression Analysis Predicting App Users versus App Nonusers (n = 20008)

| **IV** | **Estimate** | **SE** | ***z*** | ***P*** | **OR** | **95%CI** |
| --- | --- | --- | --- | --- | --- | --- |
| Age^a^ |  |  |  |  |  |  |
| 30-44 | -0.139 | 0.064 | -2.164 | .030 | 0.870 | [0.768, 0.987] |
| 45-59 | -0.307 | 0.065 | -4.750 | < .001 | 0.736 | [0.648, 0.835] |
| ≥ 60 | -0.562 | 0.068 | -8.281 | < .001 | 0.570 | [0.499, 0.651] |
| Gender^b^ | -0.184 | 0.040 | -4.556 | < .001 | 0.832 | [0.769, 0.900] |
| BMI | 0.018 | 0.005 | 3.506 | < .001 | 1.018 | [1.008, 1.028] |
| Married | 0.048 | 0.050 | 0.961 | .336 | 1.049 | [0.952, 1.157] |
| One or more children | 0.145 | 0.051 | 2.865 | .004 | 1.156 | [1.047, 1.277] |
| Education level^c^ |  |  |  |  |  |  |
| High school | 0.147 | 0.133 | 1.107 | .268 | 1.159 | [0.898, 1.513] |
| College/vocational | 0.233 | 0.135 | 1.728 | .084 | 1.262 | [0.974, 1.654] |
| University or above | 0.424 | 0.132 | 3.212 | .001 | 1.528 | [1.186, 1.991] |
| Other | 0.818 | 0.225 | 3.641 | < .001 | 2.265 | [1.453, 3.509] |
| Job | 0.245 | 0.045 | 5.466 | < .001 | 1.277 | [1.170, 1.395] |
| Household income^d^ (JPY) |  |  |  |  |  |  |
| 3-5m | -0.084 | 0.056 | -1.506 | .132 | 0.919 | [0.824, 1.026] |
| 5-7m | 0.030 | 0.063 | 0.472 | .637 | 1.030 | [0.910, 1.166] |
| 7-10m | 0.115 | 0.067 | 1.723 | .085 | 1.122 | [0.984, 1.279] |
| ≥ 10m | 0.337 | 0.074 | 4.585 | < .001 | 1.401 | [1.213, 1.618] |
| Other | -0.232 | 0.062 | -3.736 | < .001 | 0.793 | [0.702, 0.896] |
| PA level^e^ |  |  |  |  |  |  |
| Minimally active | 0.457 | 0.044 | 10.329 | < .001 | 1.579 | [1.448, 1.723] |
| HEPA^f^ | 0.757 | 0.052 | 14.514 | < .001 | 2.132 | [1.925, 2.362] |
| Stage of change^g^ |  |  |  |  |  |  |
| Contemplation | 0.628 | 0.068 | 9.265 | < .001 | 1.875 | [1.642, 2.143] |
| Preparation | 1.064 | 0.066 | 16.067 | < .001 | 2.897 | [2.547, 3.302] |
| Action | 1.302 | 0.092 | 14.097 | < .001 | 3.676 | [3.067, 4.405] |
| Maintenance | 1.289 | 0.065 | 19.949 | < .001 | 3.628 | [3.199, 4.122] |

^a^ Reference: < 30 years.

^b^ Men = 0; Women = 1

^c^ Reference: middle school.

^d^ Reference: < 3m.

^e^ Reference: inactive.

^f^ HEPA = Health enhancing physical activity.

^g^ Reference: Precontemplation.

**Table S2**

Logistic Regression Analyses Predicting the Uses of Individual App Functions That Were Associated with Physical Activity Levels (n = 4465)

| **IV** | **Estimate** | **SE** | ***z*** | ***P*** | **OR** | **95%CI** |
| --- | --- | --- | --- | --- | --- | --- |
| **DV: Sensor Information** |  |  |  |  |  |  |
| Age^a^ |  |  |  |  |  |  |
| 30-44 | 0.100 | 0.106 | 0.941 | .347 | 1.105 | [0.898, 1.360] |
| 45-59 | 0.224 | 0.108 | 2.078 | .038 | 1.251 | [1.013, 1.546] |
| ≥ 60 | 0.139 | 0.112 | 1.245 | .213 | 1.149 | [0.923, 1.430] |
| BMI | -0.014 | 0.009 | -1.628 | .104 | 0.986 | [0.969, 1.003] |
| Married | -0.015 | 0.084 | -0.181 | .856 | 0.985 | [0.836, 1.161] |
| One or more children | -0.128 | 0.085 | -1.513 | .130 | 0.880 | [0.745, 1.038] |
| Job | -0.071 | 0.080 | -0.895 | .371 | 0.931 | [0.797, 1.088] |
| Gender^b^ | -0.179 | 0.069 | -2.591 | .010 | 0.836 | [0.730, 0.957] |
| Education level^c^ |  |  |  |  |  |  |
| College/vocational | 0.164 | 0.091 | 1.800 | .072 | 1.178 | [0.986, 1.407] |
| University or above | 0.157 | 0.076 | 2.065 | .039 | 1.170 | [1.008, 1.357] |
| Other | -0.311 | 0.305 | -1.020 | .308 | 0.733 | [0.400, 1.330] |
| Household income^d^ (JPY) |  |  |  |  |  |  |
| 3-5m | 0.204 | 0.098 | 2.093 | .036 | 1.227 | [1.013, 1.486] |
| 5-7m | 0.160 | 0.109 | 1.473 | .141 | 1.174 | [0.948, 1.453] |
| 7-10m | 0.174 | 0.114 | 1.532 | .125 | 1.190 | [0.953, 1.488] |
| ≥ 10m | 0.605 | 0.125 | 4.833 | < .001 | 1.831 | [1.434, 2.342] |
| Other | 0.054 | 0.109 | 0.501 | .617 | 1.056 | [0.853, 1.307] |
| **DV: Goal Setting** |  |  |  |  |  |  |
| Age^a^ |  |  |  |  |  |  |
| 30-44 | -0.041 | 0.121 | -0.336 | .737 | 0.960 | [0.758, 1.219] |
| 45-59 | -0.146 | 0.124 | -1.178 | .239 | 0.864 | [0.678, 1.103] |
| ≥ 60 | 0.283 | 0.126 | 2.250 | .024 | 1.327 | [1.038, 1.701] |
| BMI | 0.002 | 0.010 | 0.213 | .832 | 1.002 | [0.983, 1.021] |
| Married | -0.017 | 0.094 | -0.181 | .856 | 0.983 | [0.818, 1.182] |
| One or more children | 0.114 | 0.095 | 1.201 | .230 | 1.121 | [0.931, 1.351] |
| Job | 0.033 | 0.089 | 0.374 | .709 | 1.034 | [0.869, 1.231] |
| Gender^b^ | -0.045 | 0.078 | -0.580 | .562 | 0.956 | [0.821, 1.113] |
| Education level^c^ |  |  |  |  |  |  |
| College/vocational | 0.095 | 0.103 | 0.927 | .354 | 1.100 | [0.899, 1.345] |
| University or above | 0.054 | 0.086 | 0.633 | .527 | 1.056 | [0.893, 1.250] |
| Other | -0.122 | 0.355 | -0.344 | .731 | 0.885 | [0.422, 1.721] |
| Household income^d^ (JPY) |  |  |  |  |  |  |
| 3-5m | 0.077 | 0.110 | 0.693 | .488 | 1.080 | [0.870, 1.341] |
| 5-7m | 0.203 | 0.122 | 1.666 | .096 | 1.225 | [0.965, 1.557] |
| 7-10m | 0.160 | 0.128 | 1.248 | .212 | 1.173 | [0.913, 1.507] |
| ≥ 10m | 0.457 | 0.133 | 3.441 | .001 | 1.579 | [1.217, 2.049] |
| Other | -0.223 | 0.129 | -1.721 | .085 | 0.800 | [0.620, 1.030] |
| **DV: Goal Progress** |  |  |  |  |  |  |
| Age^a^ |  |  |  |  |  |  |
| 30-44 | -0.070 | 0.144 | -0.486 | .627 | 0.932 | [0.704, 1.239] |
| 45-59 | 0.321 | 0.140 | 2.295 | .022 | 1.378 | [1.050, 1.817] |
| ≥ 60 | 0.804 | 0.142 | 5.673 | < .001 | 2.234 | [1.697, 2.959] |
| BMI | 0.019 | 0.010 | 1.907 | .057 | 1.020 | [0.999, 1.040] |
| Married | -0.098 | 0.100 | -0.974 | .330 | 0.907 | [0.745, 1.104] |
| One or more children | -0.013 | 0.102 | -0.132 | .895 | 0.987 | [0.809, 1.206] |
| Job | -0.094 | 0.094 | -1.000 | .317 | 0.910 | [0.758, 1.095] |
| Gender^b^ | 0.111 | 0.084 | 1.322 | .186 | 1.118 | [0.947, 1.318] |
| Education level^c^ |  |  |  |  |  |  |
| College/vocational | -0.026 | 0.111 | -0.229 | .819 | 0.975 | [0.783, 1.212] |
| University or above | 0.066 | 0.093 | 0.712 | .477 | 1.068 | [0.892, 1.282] |
| Other | -0.039 | 0.371 | -0.105 | .916 | 0.962 | [0.442, 1.919] |
| Household income^d^ (JPY) |  |  |  |  |  |  |
| 3-5m | 0.159 | 0.118 | 1.350 | .177 | 1.172 | [0.931, 1.478] |
| 5-7m | 0.030 | 0.136 | 0.225 | .822 | 1.031 | [0.790, 1.344] |
| 7-10m | 0.332 | 0.137 | 2.431 | .015 | 1.394 | [1.066, 1.822] |
| ≥ 10m | 0.328 | 0.145 | 2.258 | .024 | 1.388 | [1.044, 1.844] |
| Other | -0.151 | 0.137 | -1.100 | .271 | 0.860 | [0.656, 1.124] |
| **DV: Journaling** |  |  |  |  |  |  |
| Age^a^ |  |  |  |  |  |  |
| 30-44 | 0.010 | 0.130 | 0.078 | .938 | 1.010 | [0.784, 1.306] |
| 45-59 | -0.241 | 0.136 | -1.772 | .076 | 0.790 | [0.602, 1.027] |
| ≥ 60 | -0.242 | 0.143 | -1.691 | .091 | 0.790 | [0.594, 1.040] |
| BMI | -0.006 | 0.011 | -0.505 | .613 | 0.990 | [0.972, 1.016] |
| Married | -0.151 | 0.110 | -1.369 | .171 | 0.860 | [0.693, 1.067] |
| One or more children | 0.125 | 0.110 | 1.129 | .259 | 1.130 | [0.914, 1.408] |
| Job | 0.127 | 0.107 | 1.180 | .238 | 1.130 | [0.920, 1.402] |
| Gender^b^ | -0.220 | 0.090 | -2.439 | .015 | 0.800 | [0.672, 0.957] |
| Education level^c^ |  |  |  |  |  |  |
| College/vocational | 0.158 | 0.125 | 1.263 | .207 | 1.170 | [0.916, 1.495] |
| University or above | 0.321 | 0.101 | 3.168 | .002 | 1.380 | [1.132, 1.684] |
| Other | -0.111 | 0.450 | -0.246 | .805 | 0.890 | [0.334, 2.012] |
| Household income^d^ (JPY) |  |  |  |  |  |  |
| 3-5m | 0.301 | 0.134 | 2.243 | .025 | 1.350 | [1.040, 1.762] |
| 5-7m | 0.547 | 0.143 | 3.838 | < .001 | 1.730 | [1.309, 2.289] |
| 7-10m | 0.245 | 0.153 | 1.599 | .110 | 1.280 | [0.947, 1.725] |
| ≥ 10m | 0.669 | 0.154 | 4.334 | < .001 | 1.950 | [1.443, 2.644] |
| Other | -0.125 | 0.163 | -0.767 | .443 | 0.880 | [0.640, 1.212] |
| **DV: GPS/Map** |  |  |  |  |  |  |
| Age^a^ |  |  |  |  |  |  |
| 30-44 | 0.336 | 0.146 | 2.299 | .021 | 1.399 | [1.054, 1.869] |
| 45-59 | 0.287 | 0.149 | 1.934 | .053 | 1.333 | [0.999, 1.789] |
| ≥ 60 | 0.288 | 0.155 | 1.850 | .064 | 1.333 | [0.986, 1.813] |
| BMI | -0.001 | 0.011 | -0.060 | .952 | 0.999 | [0.977, 1.021] |
| Married | -0.364 | 0.112 | -3.246 | .001 | 0.695 | [0.558, 0.866] |
| One or more children | 0.173 | 0.114 | 1.519 | .129 | 1.189 | [0.952, 1.490] |
| Job | -0.028 | 0.109 | -0.259 | .796 | 0.972 | [0.786, 1.204] |
| Gender^b^ | -0.523 | 0.095 | -5.503 | < .001 | 0.593 | [0.492, 0.713] |
| Education level^c^ |  |  |  |  |  |  |
| College/vocational | 0.208 | 0.123 | 1.688 | .091 | 1.232 | [0.966, 1.568] |
| University or above | 0.047 | 0.103 | 0.459 | .646 | 1.048 | [0.858, 1.284] |
| Other | -0.024 | 0.423 | -0.056 | .955 | 0.977 | [0.392, 2.110] |
| Household income^d^ (JPY) |  |  |  |  |  |  |
| 3-5m | -0.060 | 0.134 | -0.443 | .658 | 0.942 | [0.724, 1.227] |
| 5-7m | 0.214 | 0.144 | 1.490 | .136 | 1.239 | [0.935, 1.642] |
| 7-10m | 0.077 | 0.153 | 0.501 | .616 | 1.080 | [0.800, 1.456] |
| ≥ 10m | 0.429 | 0.156 | 2.760 | .006 | 1.536 | [1.132, 2.084] |
| Other | -0.133 | 0.155 | -0.856 | .392 | 0.875 | [0.644, 1.185] |
| **DV: Energy analysis** |  |  |  |  |  |  |
| Age^a^ |  |  |  |  |  |  |
| 30-44 | 0.087 | 0.127 | 0.685 | 0.493 | 1.091 | [0.851, 1.403] |
| 45-59 | -0.201 | 0.133 | -1.510 | 0.131 | 0.818 | [0.630, 1.063] |
| ≥ 60 | -0.091 | 0.139 | -0.657 | 0.511 | 0.913 | [0.697, 1.199] |
| BMI | 0.006 | 0.011 | 0.583 | 0.560 | 1.006 | [0.985, 1.027] |
| Married | -0.073 | 0.105 | -0.697 | 0.486 | 0.929 | [0.756, 1.143] |
| One or more children | 0.065 | 0.105 | 0.619 | 0.536 | 1.067 | [0.869, 1.313] |
| Job | 0.173 | 0.102 | 1.694 | 0.090 | 1.189 | [0.974, 1.454] |
| Gender^b^ | -0.085 | 0.086 | -0.984 | 0.325 | 0.918 | [0.775, 1.088] |
| Education level^c^ |  |  |  |  |  |  |
| College/vocational | 0.169 | 0.117 | 1.438 | 0.150 | 1.184 | [0.940, 1.490] |
| University or above | 0.254 | 0.097 | 2.618 | 0.009 | 1.289 | [1.067, 1.561] |
| Other | -0.746 | 0.531 | -1.405 | 0.160 | 0.474 | [0.141, 1.195] |
| Household income^d^ (JPY) |  |  |  |  |  |  |
| 3-5m | 0.232 | 0.127 | 1.825 | 0.068 | 1.262 | [0.984, 1.622] |
| 5-7m | 0.305 | 0.139 | 2.200 | 0.028 | 1.356 | [1.034, 1.781] |
| 7-10m | 0.333 | 0.143 | 2.321 | 0.020 | 1.395 | [1.054, 1.849] |
| ≥ 10m | 0.532 | 0.149 | 3.571 | 0.000 | 1.702 | [1.272, 2.280] |
| Other | -0.051 | 0.150 | -0.339 | 0.735 | 0.950 | [0.707, 1.274] |

^a^ Reference: < 30 years.

^b^ Men = 0; Women = 1.

^c^ Reference: middle school.

^d^ Reference: < 3m.
